# Effects of PMA (PHORBOL-12-MYRISTATE-13-ACETATE) on the Developing Rodent Brain

**DOI:** 10.1155/2015/318306

**Published:** 2015-03-30

**Authors:** Mark Dzietko, Maria Hahnemann, Oliver Polley, Marco Sifringer, Ursula Felderhoff-Mueser, Christoph Bührer

**Affiliations:** ^1^Department of Pediatrics I, University Hospital Essen, University of Duisburg-Essen, 45147 Essen, Germany; ^2^Department of Radiology, University Hospital Essen, University of Duisburg-Essen, 45147 Essen, Germany; ^3^Department of Neonatology, Charité - Universitätsmedizin Berlin, 13353 Berlin, Germany; ^4^Department of Anesthesiology and Intensive Care Medicine, Charité - Universitätsmedizin Berlin, 13353 Berlin, Germany

## Abstract

Perinatal infections have a negative impact on brain development. However, the underlying mechanisms leading to neurological impairment are not completely understood and reliable models of inflammation are urgently needed. Using phorbol-myristate-acetate as an activator of inflammation, we investigated the effect on the developing rodent brain. Neonatal rats and mice deficient in IL-18 or IRAK-4 were exposed to PMA. Brains were assessed for regulation of pro- and anti-inflammatory cytokines and cell death 24 hrs, 7 and 14 days after treatment. PMA induced an inflammatory response and caused widespread neurodegeneration in the brains of 3- and 7-day-old rats. In contrast, 14-day-old rats were resistant to the neurotoxic effect of PMA. Histological evaluation at the age of 14 and 21 days revealed a destruction of the cortical microstructure with decreased numerical density of neuronal cells. Mice deficient in IL-18 or IRAK-4 were protected against PMA induced brain injury. PMA treatment during a vulnerable period can alter brain development. IL-18 and IRAK-4 appear to be important for the development of PMA induced injury.

## 1. Introduction

Recent advances in the understanding of fetal physiology and neonatal intensive care medicine have resulted in markedly increased survival rates of premature infants. However, severe motor and cognitive impairment still affects a considerable proportion of surviving patients, who exhibit disturbances in learning, cognition, and attention [1]. Whereas many of these disabilities have been attributed mostly to white matter injury, disrupted grey matter development also appears to contribute to disabilities of preterm survivors [2]. Brain injury with widespread loss of neurons has been noted in the cortex, hippocampus, and thalamus of postmortem specimens from patients with periventricular leukomalacia (PVL) [3]. However, the pathology of perinatal brain injury is complex and depends on injury mode and developmental stage [4]. Over the last decade the hypoxic-ischemic paradigm for neonatal brain injury has been replaced by a multifactorial hypothesis which recognizes the key role elevated levels of proinflammatory cytokines play in systemic inflammation of the brain [5]. In previous studies, we induced inflammation by hyperoxia in combination with lipopolysaccharide (LPS) and identified the role of inflammatory cytokines in neurodegenerative processes in the developing brain [6, 7]. These inflammatory mediators trigger strong alterations in brain development and changes in microstructural integrity [7]. Furthermore, an increase in degenerating cells in the thalamus of newborn rabbits prenatally exposed to endotoxins suggests impairment of grey matter [8].

T-cell activation is a fundamental step for an effective immune response and is triggered by the interaction of receptors to specific ligands with concomitant formation of diacylglycerol and activation of protein kinase C (PKC). PMA, a structural analogue of diacylglycerol, is a potent activator of PKC [9]. Activation of PKC by PMA results in the induction of granulocytes, proinflammatory cytokines like IL-6, TNF-*α*, IL-1*β*, increased levels of glutamate, and the release of reactive oxygen species [10, 11]. In the developing rodent brain, IL-1*β* and IL-18 play a pivotal role in inflammatory responses with subsequent cell death [6, 7, 12]. Engagement of IL-1*β* and IL-18 receptors initiates a common intracellular signaling cascade wherein the myeloid differentiation factor (MyD88) and TNF receptor associated factor 6 (TRAF6) serve as key adaptor proteins. It has been shown that IRAK-4 mediates signaling between MyD88 and TRAF6 thereby providing an essential component in promoting downstream signals. Nevertheless the complex mechanisms of inflammation leading to brain injuries are still controversial and remain unclear. Seeking to better understand the histological injury patterns and pathways involved in inflammatory neonatal brain injuries, we applied PMA as an inflammatory trigger to neonatal rodents. By administering PMA to IRAK-4 and IL-18 knockout mice we provide additional evidence that disruption of inflammatory pathways is sufficient to protect the developing brain from PMA induced injury.

## 2. Materials and Methods

### 2.1. Animals and Drug Administration

All animal experiments were carried out in accordance with institutional guidelines and conform to the European Guidelines for Use of Experimental Animals by FELASA (Federation of European Laboratory Animal Science Association) and were approved by the local animal research committee. 2-, 6- and 13-day-old randomly assigned male Wistar rat pups (BgVV, Berlin, Germany) received a single intraperitoneal (i.p.) injection of vehicle (normal saline, Braun, Ingelheim, Germany) or PMA (500 *μ*g/kg, Sigma, St. Louis, USA). A subgroup of 6-day-old rat pups received a single injection of vehicle or different PMA doses (100, 200, and 500 *μ*g/kg). Animals were sacrificed 24 hours or 7 and 14 days following PMA exposure.

6-day-old homozygous IRAK-4-deficient C57BL/6 mice (provided by Dr. Yeh, Ontario Cancer Institute, Toronto, Canada) and Interleukin-18-deficient C57BL/6 mice (B6.129P2-Il18tm1Aki/J, Jackson Laboratory, Bar Harbor, USA) received a single injection of PMA (200 *μ*g/kg). Wild-type mice serving as controls received either PMA or vehicle. All pups were kept with their dams except for injections to avoid hypothermia and malnutrition.

### 2.2. Tissue Sampling

Animals subjected to histological analysis received an overdose of chloral-hydrate and were transcardially perfused with 0.1 M phosphate-buffered saline followed by 4% paraformaldehyde in cacodylate buffer solution. Coronal paraffin sections (10 *μ*m, microtome HM 360; Microm, Giessen, Germany) were stained with cresyl violet to determine total cell density in P14 and P21 animals. For real-time PCR analysis, animals were decapitated, brains were immediately removed, and hemispheres were snap frozen in liquid nitrogen and stored at −80°C until analysis.

### 2.3. RNA Extraction and Semiquantitative Real-Time PCR

Total RNA was isolated from snap-frozen tissue by acidic phenol/chloroform extraction (peqGOLD RNAPure; PEQLAB Biotechnologie, Erlangen, Germany) and 2 *μ*g of RNA was reverse transcribed. The PCR products of IL-1*β*, TNF-*α*, TGF-*β*1, and hypoxanthine-guanine phosphoribosyltransferase (HPRT, as internal standard) were quantified in real time, using dye-labeled fluorogenic reporter oligonucleotide probes and primers (Metabion, Munich, Germany) with the following sequences and corresponding GenBank accession numbers:* IL-1β* (NM_031512) sense 5′-AACAAAAATGCCTCGTGCTGTCT-3′, antisense 5′-TGTTGGCTTATGTTGTGTCCATTG-3′, probe 5′-ACCCATGTGAGCTGAAAGCTCTCC-3′;* TNF-α* (NM_012675) sense 5′-TCGAGTGACAAGCCCGTAGC-3′, antisense 5′-CTCAGCCACTCCAGCTGCTC-3′, probe 5′-CGTCGTAGCAAACCACCAAGCAGA-3′;* TGF-β1* (NM_021578) sense 5′-CCCTGCCCCTACATTTGGA-3′, antisense 5′-ACGGTGATGCGGAAGCAC-3′, probe 5′-CACACAGTACAGCAAGGTCCTTGCCCT-3′;* HPRT* (NM_012583) sense 5′-GGAAAGAACGTCTTGATTGTTGAA-3′, antisense 5′-CCAACACTTCGAGAGGTCCTTTT-3′, probe 5′-CTTTCCTTGGTCAAGCAGTACAGCCCC-3′. All probes were labeled at their 5′ ends with the reporter dye 6-carboxy-fluoresceine (FAM) and at their 3′ ends with the quencher dye 6-carboxy-tetramethylrhodamine (TAMRA). Real-time PCR and detection were performed in triplicate and repeated 3 times for each sample using a total reactive volume of 13 *μ*L which contained 6.5 *μ*L of 2x TaqMan Universal PCR Master Mix (Applied Biosystems, Foster City, USA), 2.5 *μ*L of 1.25 *μ*M oligonucleotide mix, 0.5 *μ*L (0.5 *μ*M) of probe, and 50 ng of cDNA template. The PCR amplification was performed in 96-well optical reaction plates for 40 cycles with each cycle at 94°C for 15 s and 6°C for 1 min. The expression of IL-1*β*, TNF-*α*, TGF-*β*1, and HPRT was analyzed with the real-time PCR ABI Prism 7500 sequence detection system (Applied Biosystems) according to the 2^−ΔΔCT^ method [13].

### 2.4. De Olmos Cupric Silver Staining

Brains were embedded in agar; coronal sections (70 *μ*m) were cut and processed for staining with silver and cupric nitrate according to the method of De Olmos and Ingram [14]. Degenerating cells were identified by a distinct dark appearance due to silver impregnation.

### 2.5. Fluoro-Jade B Staining

Paraffin sections were incubated for 10 min in 0.06% solution of potassium permanganate and stained for 20 min in 0.0004% solution of fluoro-jade B (Histo-Chem, Jefferson, USA) dissolved in 0.1% acetic acid. Sections were coverslipped with nonfluorescent mounting media (Sigma) and samples were examined with an epifluorescent microscope using blue light (excitation 450–490 nm, emission 515–565 nm). Degenerating cells were identified by a green fluorescent appearance.

### 2.6. TUNEL Staining

Staining was performed on paraffin sections using the Apop Tag Peroxidase kit (Oncor Appligene, Heidelberg, Germany) according to the manufacturer's instructions. Sections were finally stained with diaminobenzidine (Sigma) and TUNEL positive cells were identified by brown appearance.

### 2.7. Immunohistochemistry for Cleaved Caspase-3

Paraffin sections were microwaved in 10 mM citrate buffer and endogenous peroxidase activity was blocked with 0.6% hydrogen peroxide. Slices were incubated with normal goat serum overnight at 4°C with anti-cleaved caspase-3 antibody (1 : 100; Cell Signaling, Frankfurt, Germany). Sections were treated with rabbit anti-goat IgG. After detection with ABC kit (Vector Laboratories, Peterborough, UK), positive cells were visualized with diaminobenzidine.

### 2.8. Quantification of Neurodegeneration

Degenerating cells were identified by using De Olmos, cresyl violet (rat), or fluoro-jade B staining (mice). Mean numerical cell densities (*N*_*v*_) of degenerating cells or total cell density (cells/mm^3^) were acquired in 17 brain regions by means of a stereological dissector. An unbiased counting frame (0.05 × 0.05 mm, dissector height 0.01 mm for fluoro-jade B/cresyl violet, 0.07 mm for silver staining) and a high aperture objective were used for sampling. Numerical densities (*N*_*v*_) within each brain region for cell degeneration or total cell density were determined with eight dissectors [15]. The generated cumulative score for each animal was used for comparison.

### 2.9. Statistical Analyses

Values are presented as mean ± SEM. Comparisons among groups were made using one-way analysis of variance (ANOVA) with Newman-Keul post hoc test or Student's *t*-test as appropriate. Statistical significance was determined at *P* < 0.05.

## 3. Results

### 3.1. PMA Exposure Affects Postnatal Weight Gain

Han Wistar rats treated with PMA (500 *μ*g/kg) showed impaired weight gain compared to control littermates. Average weight gain was significantly reduced in animals 7 and 14 days old. Animals receiving a lower dose or are 3 days old showed adequate weight gain. Survival rate was not altered within treatment groups (Table 1).

### 3.2. PMA Triggers mRNA Expression of IL-1*β*

**Figure 1:**
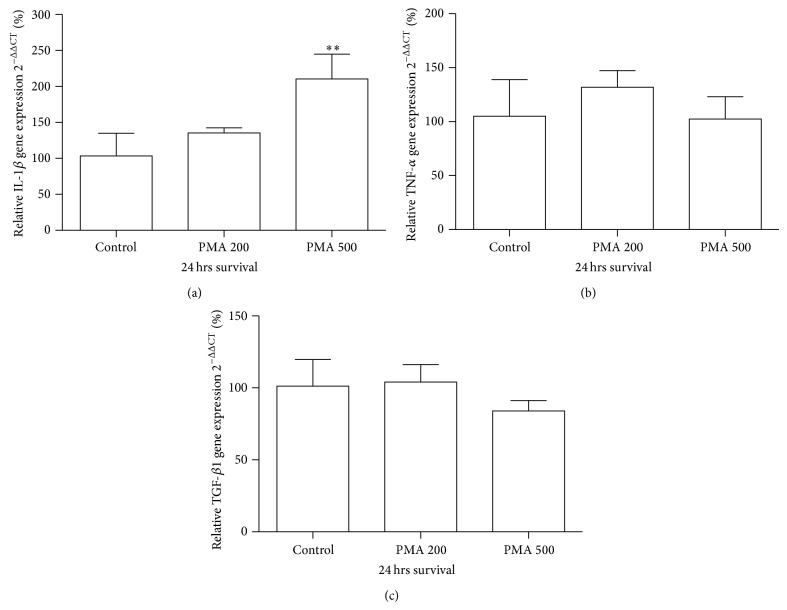
Increased expression of proinflammatory cytokine IL-1*β* in PMA (500 *μ*g/kg) treated rat pups. ((a)–(c)) Quantitative analysis of IL-1*β*, TNF-*α*, and TGF-*β*1 mRNA expression at 24 h after PMA administration. Data are normalized to levels of vehicle treated animals (control = 100%). Values are presented as mean ± SEM. ^∗∗^ *P* < 0.01, one-way analysis of variance (ANOVA) with Newman-Keul post hoc test.

To determine how the proinflammatory cytokines IL-1*β* and TNF-*α* and the anti-inflammatory cytokine TGF-*β*1 were affected by PMA exposure in the neonatal brain, we performed real-time polymerase chain reaction (PCR) on mRNA isolated from brain hemispheres obtained 24 hours after exposure. Quantitative analysis of mRNA expression revealed a significant increase of IL-1*β* (Figure 1(a)) after PMA exposure (500 *μ*g/kg). TNF-*α* and TGF-*β*1 expression was not affected by PMA therapy (Figures 1(b) and 1(c)). Control animals and pups treated with a lower dose (200 *μ*g/kg) exhibited no modification of cytokine expression (Figures 1(a)–1(c)).

### 3.3. PMA Induces a Robust Neurodegenerative Response in the Immature Brain

**Figure 2:**
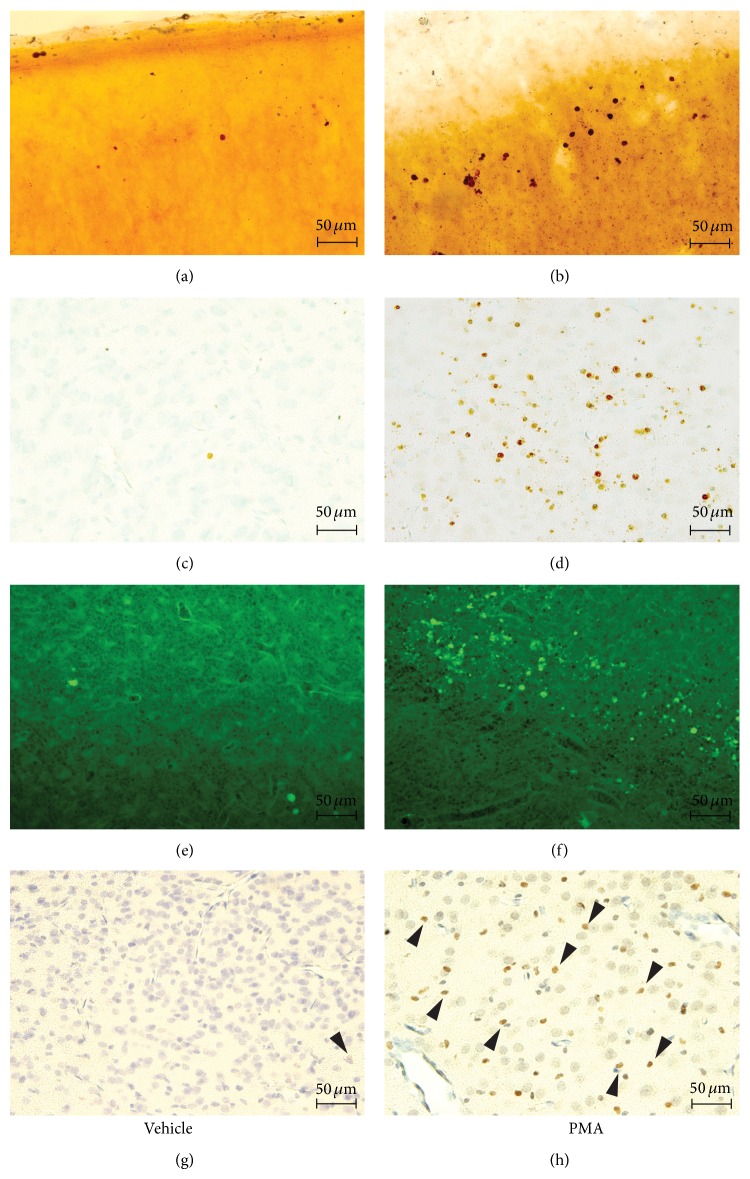
PMA induced cell death in the immature rat brain. Representative microscopic overviews ((a)–(h), 20x) of affected brain regions. In the injured frontal cortex from an animal subjected to PMA (500 *μ*g/kg), degenerating cells appear as small dark dots in 70 *μ*m silver-stained sections (b), TUNEL staining performed in 10 *μ*m-thick cortical sections of the ventral thalamus confirms DNA fragmentation within degenerating cells (d). Fluoro-Jade B staining of the laterodorsal thalamus depicting neurodegenerative changes in P7 rats 24 hours after PMA exposure (f). Immunohistochemistry demonstrates caspase-3-immunopositive cells in the laterodorsal thalamus of a 7-day-old rat subjected to PMA ((h), black arrowheads). Photomicrographs of control animals ((a), (c), (e), (g)) showed no neurodegenerative response.

**Figure 3:**
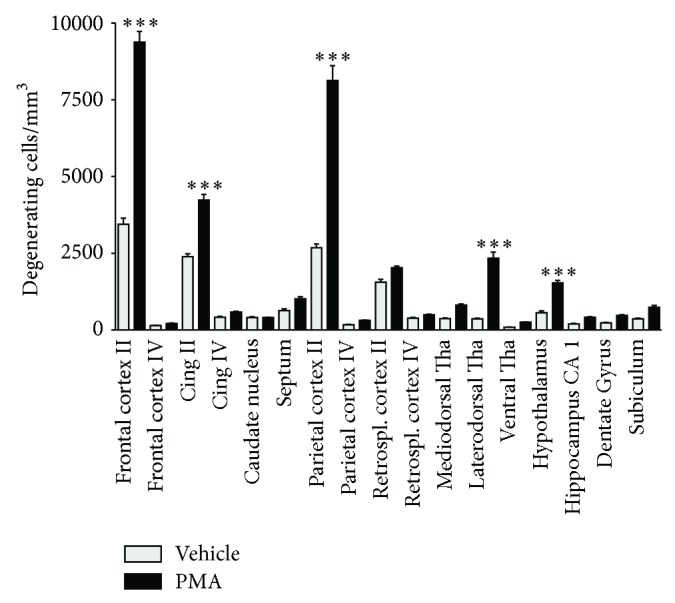
Graphic illustration of PMA (500 *μ*g/kg) induced neurodegeneration in 17 different brain regions of 7-day-old rats. Degenerating neurons were visualized by De Olmos cupric silver staining and sampled by the optical dissector method to provide a numerical density (degenerating cells/mm^3^). The numerical density counts were added to give a cumulative score for degenerating cells for each brain region, and a mean was calculated for each treatment condition (*n* = 8–10 per group). Values are presented as mean ± SEM. ^∗∗∗^ *P* < 0.001, one-way analysis of variance (ANOVA) with Newman-Keul post hoc test.

To explore whether a single injection of PMA exerted neurodegenerative effects, P6 rat pups were exposed to 500 *μ*g/kg PMA. Histological stainings revealed widespread neurodegeneration (Figures 2(b), 2(d), 2(f), and 2(h)). In age-matched littermates receiving vehicle, degenerating cells were sparse (Figures 2(a), 2(c), 2(e), and 2(g)). Fluoro-jade B, TUNEL, and caspase-3 staining showed a similar distribution pattern of degenerating cells compared to silver staining, suggesting apoptosis as a mechanism of PMA induced cell death. The primary affected brain regions were the second layer of the cortex, thalamus, and hypothalamus, as depicted in Figure 3.

### 3.4. High Dose PMA Induces Neurodegeneration in the Developing Brain

**Figure 4:**
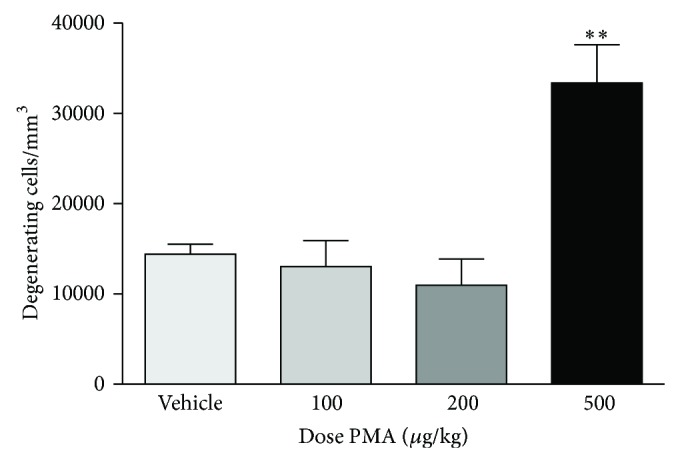
Graphic illustration of neurodegeneration in 7-day-old rats following different doses of PMA (100, 200, and 500 *μ*g/kg). Degenerating neurons were visualized by De Olmos cupric silver staining and sampled by the optical dissector method to provide a numerical density (degenerating cells/mm^3^) for different brain regions. The numerical density counts from these brain regions were added to give a cumulative score for degenerating cells for each brain, and a mean was calculated for each treatment condition (*n* = 7–10 per group). Values are presented as mean ± SEM. ^∗∗^ *P* < 0.01, one-way analysis of variance (ANOVA) with Newman-Keul post hoc test.

To Investigate the effects of different doses of PMA on the immature brain 6-day-old rats were treated with 100, 200, or 500 *μ*g/kg PMA. Animals treated with lower doses of PMA were indistinguishable from control animals. However, profound neurodegeneration was confirmed after treatment with 500 *μ*g/kg PMA (Figure 4).

### 3.5. PMA-Induced Neurodegeneration Is Age Dependent

**Figure 5:**
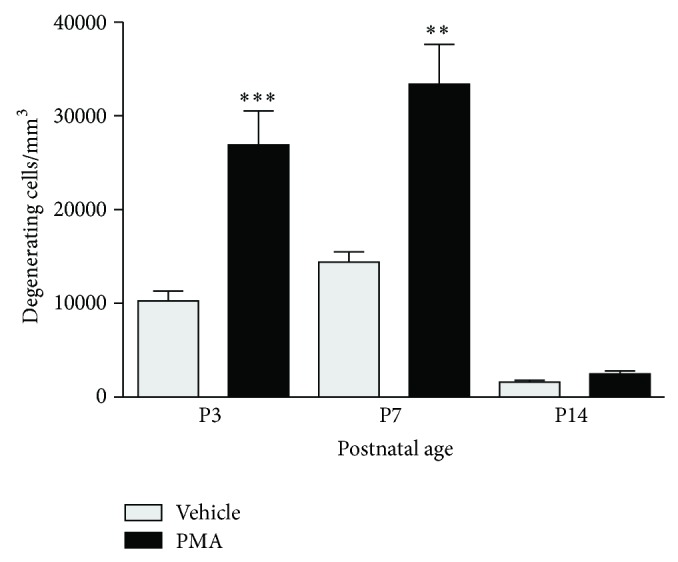
Age dependency of PMA (500 *μ*g/kg) induced neurodegeneration using De Olmos cupric silver staining. The numerical density counts from brain regions were added to give a cumulative score for degenerating cells for each brain, and a mean was calculated for each age group (*n* = 8–14 per group). Values are presented as mean ± SEM. ^∗∗^ *P* < 0.01; ^∗∗∗^ *P* < 0.001; one-way analysis of variance (ANOVA) with Newman-Keul post hoc test.

To determine whether the apoptotic response to PMA may differ as a function of developmental age, we subjected 2-, 6-, and 13-day-old rats to PMA (500 *μ*g/kg). P3 and P7 animals exposed to PMA revealed enhanced neurodegeneration in comparison to controls suggesting a time window between P3 and P7 when neuronal cells are vulnerable to PMA-induced cell death. In contrast, P14 rats showed no significant difference after treatment with PMA in comparison to controls (Figure 5).

### 3.6. Neonatal PMA Exposure Reduces Neuronal Cell Density in the Adolescent Rat

**Figure 6:**
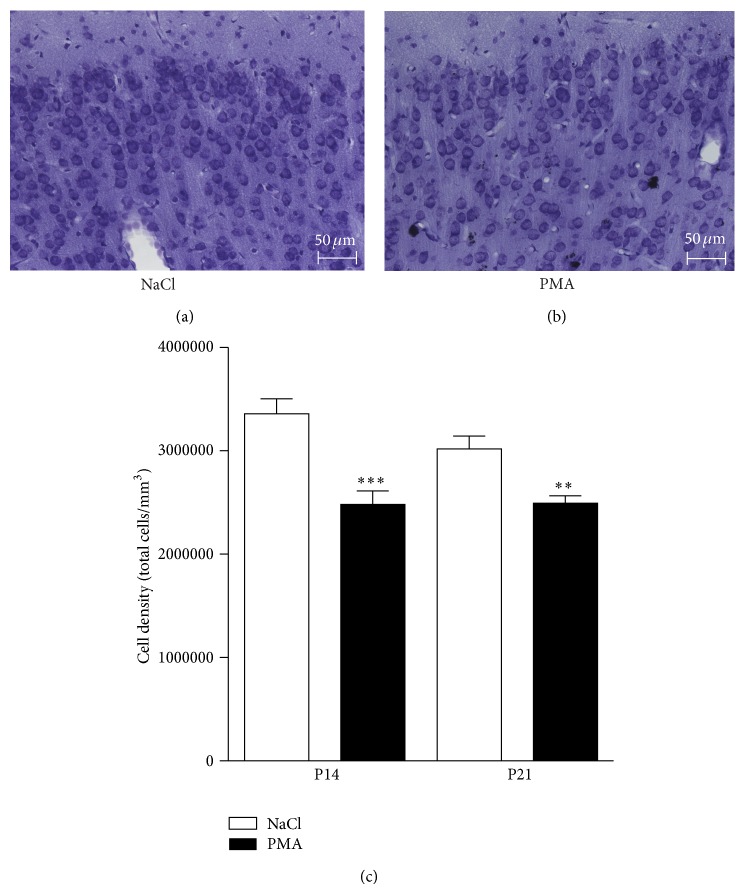
Cresyl violet staining (magnification 20x) performed in 10 *μ*m-thick sections depicting disorganized cytoarchitecture of layers I and II of the frontal cortex in 14-day-old rats subjected to vehicle (a) or PMA (500 *μ*g/kg) (b) at P6. 14- and 21-day-old rat pups treated with PMA at P6 showed reduced total cell density in comparison with controls (c) (*n* = 7–11 per group). Values are presented as mean ± SEM. ^∗∗^ *P* < 0.01; ^∗∗∗^ *P* < 0.001, one-way analysis of variance (ANOVA) with Newman-Keul post hoc test.

In order to investigate whether neonatal exposure to PMA diminishes the total amount of neuronal cells and affects cortical cytoarchitecture in older animals, we administered PMA to 6-day-old rats. Histological evaluation with cresyl violet staining at the age of 14 and 21 days revealed a destruction of the cortical microstructure (Figures 6(a) and 6(b)), with a decreased numerical density of neuronal cells (Figure 6(c)). The cytoarchitecture in animals exposed to PMA as a neonate was disorganized, lacking the expected arrangement of neurons on columns typically seen in the normal neocortex. These results suggest a long-lasting effect on brain development by PMA exposure early in life.

### 3.7. Mice Deficient in IRAK-4 and IL-18 Are Protected against PMA-Induced Cell Death

**Figure 7:**
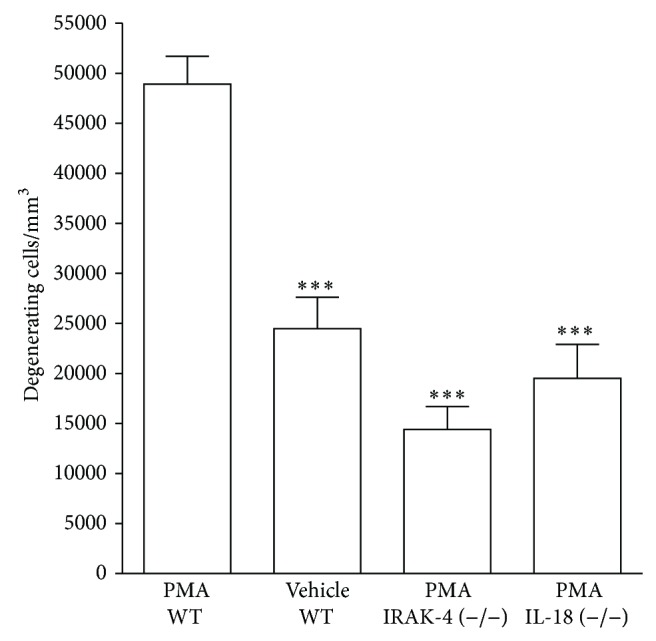
Mice deficient in IRAK-4 or IL-18 are protected against PMA-induced neurodegeneration. Six-day-old homozygous IRAK-4 and IL-18 mice were subjected to PMA (200 *μ*g/kg). Quantification of cell death was performed on fluoro-jade B-labeled sections. IL-18 (−/−) (*n* = 4) and IRAK-4 (−/−) (*n* = 9) deficient mice displayed significant differences in degenerating cells compared with their wild-type littermates (*n* = 6). PMA induced a strong neurodegenerative response in wild-type mice compared to their vehicle treated littermates. Values are presented as mean ± SEM. ^∗∗∗^ *P* < 0.001, one-way analysis of variance (ANOVA) with Newman-Keul post hoc test.

To confirm a functional contribution of IL1-*β* and IL-18 in PMA-induced cell death, we injected PMA (200 *μ*g/kg) to 6-day-old IRAK-4 and IL-18 knockout mice. Wild-type mice treated with PMA or vehicle served as controls. Mice receiving a higher dose of PMA (500 *μ*g/kg) did not survive suggesting a higher sensitivity of mice to PMA. Brains were processed for fluoro-jade B staining 24 hours later to detect neuronal injury. PMA caused neuronal cell death in the wild-type mice with a pattern very similar seen in rats. Vehicle treated wild-type animals revealed a significant difference compared to PMA treated littermates. Quantification of cell death in IRAK-4 and IL-18 knockout mice after PMA exposure displayed a strong neuroprotective effect compared to PMA treated wild-type controls (Figure 7).

## 4. Discussion

Here we describe an activation of the proinflammatory cytokine IL-1*β* and a strong neurodegenerative response in neonatal rodents after systemic PMA exposure. A single injection of PMA was sufficient to double the amount of degenerating cells. The injury pattern was confirmed in different histological stainings suggesting programmed cell death as a major component of the injury. Impairment of weight gain seems not to play a pivotal role in PMA induced neurotoxicity because older animals were not affected despite the fact that weight gain was significantly depressed. Furthermore, we found the neurotoxic effect of PMA to be confined to the first postnatal week of life. The first postnatal week of life is characterized by rapid brain growth and these results confirm the vulnerability of the neonatal brain during this growth spurt [16]. PMA treatment predominantly affected the second cortical layer and the laterodorsal thalamus but all investigated areas exhibited degenerated cells. Prenatal endotoxin exposure in rabbits revealed similar results with a profound decrease of neurons in the anterior thalamus [8]. In addition, neurodegeneration in the thalamus is combined with a decrease of dendritic arborization in the retrosplenial cortex suggesting an increased vulnerability of this important relay station [8].

From human data, it is known that the thalamus is commonly affected in patients with cerebral palsy, and atrophy of deep grey matter has previously been described in patients with PVL [2]. This contrasts to hypoxia-ischemia in a preterm ovine model, which also revealed an immature dendritic arborization without detected neuronal cell death, underlining the specific role of inflammation in neonatal brain injury and the importance of the mode of injury [17]. Interestingly, animals treated with PMA had a lower total density of neuronal cells with transformation of the cortical microarchitecture 14 and 21 days after birth. Normal cortex exhibits a specific cytoarchitecture and is horizontally organized into laminae and vertically into groups of synaptically linked cells, called neocortical minicolumns. This represents the basic processing units of the mature neocortex [18]. The cortical cytoarchitecture in animals treated with PMA early in life was disorganized, primarily affecting layer II by disrupting the expected arrangement of cells in layers and columns. Analog to our results, inflammation during the critical period of brain development induced by LPS leads to amplified neuronal injury and impairment of synaptic plasticity [19]. Furthermore, neonatal exposure of LPS and oxygen revealed decreased numbers of oligodendrocytes in the adult cortex and hippocampus [20].

PMA has been described to activate granulocytes, to elevate levels of proinflammatory cytokines like IL-6, TNF-*α*, and IL-1*β*, and to release reactive oxygen species and glutamate as potential mechanisms of injury [10, 11, 21]. In our model, we also found a strong increase of IL-1*β* as a response after PMA exposure, supporting the idea of using PMA as an activator for a systemic inflammatory response. In addition, we used IRAK-4 and IL-18 knockout mice to further address possible mechanisms involved in PMA induced neurotoxicity. IRAK-4 is required for LPS-induced activation of antigen presenting cells with IRAK-4-deficient animals being completely resistant to LPS [22, 23]. Furthermore, animals inhibited or deficient for IRAK-4 are protected against oxygen-mediated [6] and hypoxia-ischemia (HI) induced neuronal injury [24]. In our experiments IRAK-4 knockout mice treated with PMA were protected compared to their wild-type littermates. These results suggest that PMA treatment does promote IRAK-4 mediated neurodegeneration. An inflammatory role for IL-18 has been implicated in several diseases including HI in the immature brain. IL-18 is increased after HI and IL-18 deficiency has also been shown to be protective against brain injury after neonatal HI [25, 26]. In contrast, adult mice lacking the gene for IL-18 are not protected against ischemic injury emphasizing the controversy regarding the role of IL-18 in different injury models and developmental stages [27]. In our studies, mice deficient for IL-18 were protected against PMA toxicity suggesting a potential role of IL-18 in PMA triggered brain injury.

## 5. Conclusions

To our knowledge, this is the first study showing that systemic PMA application during a critical period of development can induce an inflammatory response combined with neurodegeneration and alter brain development with long-lasting effects into adolescence. The injury is characterized by diffuse grey matter injury with programmed cell death being a component involved in neuronal injury. IRAK-4 and IL-18, two major inflammatory pathways, seem to be involved in PMA triggered brain injury. The increasing problem of diffuse brain injury [28] raises in our opinion the need for more subtle models of inflammation triggered brain injury.

## Figures and Tables

**Table 1:** Average weight gain in g/day, % survival rate and number of animals used.

Age	P3		P7		P7		P7		P14	
Treatment *μ*g/kg	PMA 500	NaCl	PMA 100	NaCl	PMA 200	NaCl	PMA 500	NaCl	PMA 500	NaCl

Average weight gain g/day	1.0 ± 0.2	1.3 ± 0.1	1.0 ± 0.2	1.4 ± 0.2	1.3 ± 0.2	1.4 ± 0.2	0.5^**^ ± 0.1	1.4 ± 0.2	0.7^***^ ± 0.1	2.8 ± 0.2

% survival	100	100	88	100	100	100	91	100	100	100

*n* =	11	10	7	8	9	8	10	8	8	5

P7 and P14 rats treated with PMA 500 μg/kg showed impaired weight gain 24 hours after injection compared to their control littermates (±SEM, ^**^P < 0.01; ^∗∗∗^ *P* < 0.001, Student's *t*-test).
